# Prevalence of Work-Related Musculoskeletal Disorders: Psychological and Physical Risk Factors

**DOI:** 10.3390/ijerph18179361

**Published:** 2021-09-04

**Authors:** K. Saraswathi Krishnan, Gunasunderi Raju, Omar Shawkataly

**Affiliations:** 1 School of Distance Education, Universiti Sains Malaysia, Gelugor 11800, Malaysia; wathi_76@yahoo.com; 2 UniKL MICET Lot 1988, Kawasan Perindustrian Bandar Vendor, Taboh Naning, Alor Gajah 78000, Malaysia; oshawkataly@gmail.com

**Keywords:** prevalence works related musculoskeletal disorders, psychological risk factors, physical factors, nursing

## Abstract

Purpose—This study aimed to estimate the prevalence and risk factors of MSD pain in various anatomical regions among nurses. Method—A cross-sectional study involving a self-administered questionnaire by registered nurses with clinical experience. Data was collected using convenience sampling after obtaining informed consent. The results were drawn from a total of 300 nurses. Results—The nurses presented with occasional mental exhaustion (44.3%) and often physical exhaustion (44.0%). Almost all (97.3%) the nurses complained of having work-related pain during the last 12 months. Body parts with the most pain were the lower back (86.7%), ankles (86.7%), neck (86.0%), shoulders (85.0%), lower legs (84.7%) and upper back (84.3%). The pain frequency was rated as occasional pain for the neck and upper back, pain was often felt for the rest of the parts. Nurses complained of severe pain in the lower back (19.7%), right shoulder (29.7%) and left shoulder (30.3%). The frequency of having musculoskeletal symptoms in any body region was increased with age, lower education level, female gender, high BMI, job tenure and lifestyle. Conclusions—Nurses’ WRMSD complaints should be taken seriously to curb further risk and musculoskeletal hazards.

## 1. Introduction 

WRMSDs are the largest contributors to the occupational disease burden and are largely related to ergonomic factors found in the workplace [1]. The World Health Organization (WHO) reported that musculoskeletal conditions are the most common causes of disability and limitation related to daily living and gainful employment [2].

As evidenced in a variety of ergonomic studies, the workplace has not adapted to the changing technological advances [3]. Developed countries, such as United States, Sweden, United Kingdom and Japan, are quite concerned about safety and health since they significantly contribute to the productivity of human resources, which has an impact on a country’s economy. The issue of safety and health has become the main agenda to ensure a safe working environment and does not pose a risk to employees. These risks lead to employees working in environments that are not comfortable and therefore contribute to the high burden of WRMSDs. In studies performed in the Indian and Portuguese populations, results were obtained showing a higher prevalence of WRMSDs in the nursing workers [4,5]. 

Nurses are in great demand in most countries, regardless of whether it be for a developed country, such as the United States, or a developing country, such as Malaysia. Based on the reviewed healthcare research, the demand for nursing jobs increased simultaneously with the population in Malaysia [6]. The statistics indicate that more than 70% of the hospitals in Malaysia do not have enough nursing staff [7]. 

Long working hours with inadequate staffing (shortage) increase nurses’ risk of developing conditions, such as musculoskeletal disorder, hypertension and depression. 

Working under physical overload due to long work hours and patient handling demands leads to a high risk of developing WRMSDs [8]. Job demand is directly related to MSD development among nurses [9]. It was reported that manual patient handling is the main contribution toward WRMSDs among nurses working in hospitals [10]. Female nurses are more liable to endure WRMSDs, as they are also fulfilling responsibilities after work, including taking care of children and house chores, with insufficient rest time and a lack of exercise. Women were seen 2.1 times more often for the development of MSDs than men [11]. In general, WRMSDs hamper the working efficiency of nurses, which has an impact on patient safety in clinical practice. Due to the increasing cases of MSDs and job burnout among employees, especially among nurses, a study of the ergonomic aspects and WRMSDs toward job performance is crucial to estimate the prevalence and risk factors of MSDs among nurses.

### 1.1. Work-Related Musculoskeletal Disorders

Musculoskeletal disorders (MSDs) were identified as work-related diseases from the time of Ramazzini in the 18th century, who described classical cases of such injuries in his book [12]. Musculoskeletal disorders (MSDs) are further classified as specific or non-specific disorders. Specific musculoskeletal disorders have clear clinical features, whereas non-specific musculoskeletal disorders present with pain without evidence of a clear specific disorder. WRMSDs are known by many terms and are used interchangeably, such as repetitive motion injuries (RMIs), cumulative trauma disorders (CTDs), repetitive strain injuries (RSIs), overuse syndrome, regional musculoskeletal disorders and soft tissue disorders (STDs) [13].

### 1.2. Prevalence, Nurses and Work-Related Musculoskeletal Disorders (MSDs)

Nursing is ranked as the top occupation among all the professions that have the potential to develop MSDs, with the highest prevalence rate of MSDs in the world [14]. According to the World Health Organization (WHO), the issues of WRMSDs become worse when nurses’ daily working activities become hectic [15]. This is because this occupation has the most physically demanding jobs that involve excessive manual handling of patients and sometimes awkward postures during tasks [16]. Nurses’ work-related musculoskeletal symptoms vary depending on the wards, the hospital and even the countries they work in [4,17], which appear to be related to many different nursing occupational exposures to WRMSDs hazards.

The body regions with the greatest prevalences of WRMSDs are the low back, neck, shoulders, knees, forearms, hands and ankles/feet, although recently, the lower extremities have received more attention. MSDs may involve the upper or lower extremities and the trunk.

Studies have documented a higher annual prevalence of musculoskeletal disorders (MSDs) in at least one human body part and/or region that varied between 40 and 95% in a population of Asian nurses, and in Western populations, the low back, neck and shoulders are the most significantly affected body parts, with prevalences of 29–64%, 34–63% and 17–75%, respectively [13,18]. In contrast, a narrative literature review conducted on MSDs over the last 12 months among female nurse personnel showed that the knee and ankle/foot regions were most affected by MSDs. In the knees, the prevalence of MSDs ranged between 7.5 and 77%, and in the ankles, between 3.2 and 100%. The prevalence of MSDs in the lower legs (the shins) varied between 8.5 and 10.5%, and in the thigh/hip area, the prevalence range was 11–100% [19].

### 1.3. Psychosocial Factors

Psychosocial factors at work refer to interactions between the work environment, job content, organizational conditions and the workers’ capacities, needs, culture and personal extra-job considerations that may influence their health, work performance and job satisfaction. Nurses may need to face different types of difficulties during their work that contribute toward WRMSDs, including stress, issues with patients, issues with management and doctors [5]. A study revealed that nurses’ experiences of musculoskeletal pain are mostly related to psychosocial factors. It seems there was a positive relationship between somatic stress symptoms, including stomachache, headache, palpitations and musculoskeletal discomfort or pain. A study [20] related to psychosocial factors revealed that participants with at least one WRMSD in any of their body segments reported that they were not satisfied with their occupation and that there was a lack of cooperation between staff, poor nurse–physician interaction and a lack of support from immediate supervisors.

### 1.4. Physical Factors

Physical risk factors for WRMSDs include forces, repetition, vibrations and awkward postures. Patient handling tasks were documented as major contributing factors for WRMSDs among nursing staff. Many physical factors affect the health of nurses when performing their tasks in the hospital environment. The perceived physical demands that are commonly experienced by nurses include moving objects pulling/pushing machines, lifting patients, recurring motions and extreme flexion, bending, twisting and sudden movements [21]. These physical demands increased the risk of complaints in different body regions [18,22,23]. Other factors that are related to MSDs were being in standing positions for a prolonged time and awkward postures (during transferring patients from one location and to bed again) [24].

### 1.5. Conceptual Framework

The conceptual framework was developed based on a review of the literature, which was intended to be used as a guide.

## 2. Methods

This study employed a cross-sectional study to measure the outcome (MSD pain/discomfort) and any attribute, characteristic or exposure the nursing population experiences that increase the likelihood of developing work-related musculoskeletal disorders (WRMSDs). The presence or absence of both the exposure and the outcome (MSD pain) were determined at the same time point. The selected participants for the study were assessed for their exposures and outcomes by the investigator. The study was conducted at Hospital Sultan Abdul Halim (HSAH), Sungai Petani Kedah, Malaysia. The sampling frame for the study was drawn from all female registered nurses working in normal in-patient wards.

The selection of subjects for the study population was based on inclusion and exclusion criteria. Based on published material by [13], the prevalence of work-related musculoskeletal disorders (WRMSDs) among nurses was reported as being 73% (0.73). Using a statistical significance α of 0.05 [25], a power of 0.80 [26], precision at 0.05 and estimated prevalence of WRMSDs as 73% for an unknown population size, 303 nurses was the required sample size. Assuming an additional 10% to account for the drop-out rate, the total number of nurses required in the sample was 334. The sample size was estimated using a sample size calculation for a prevalence study without a finite population correction.

The study employed convenience sampling, where the sample for the study was obtained from six wards where the registered nurses (RNs) were working in in-patient wards with non-office hours. A total of 55 questionnaires were delivered to each ward by a researcher according to an arranged date and time frame that was agreed to by each ward nursing manager with hospital authority consent. The unit managers were contacted via a direct visit to the unit, WhatsApp and a direct telephone call at least twice weekly as a gentle reminder of the questionnaires.

The survey was carried out within 2–4 weeks’ time with a minimum of 2–3 visits to each unit (to the ward nursing manager’s office) upon official approval from the relevant authority. Each participant needed 15–20 min to complete the questionnaire. In this study, quantitative data were collected through questionnaire surveys. The survey questionnaire had 4 types of questionnaire instruments in English and a patient information sheet with an informed consent form. A pre-test was conducted among 50 registered nurses (RNs) who were working an office hours schedule (8 a.m.–5 p.m.) at the same hospital using an adapted questionnaire. The pre-test allowed the researchers to evaluate the content validity, feasibility and time requirements. Some small adaptations to the questionnaire were applied to improve the questions’ clarity and understanding. Data were analyzed using SPSS software version 13 (IBM, Armonk, NY, USA). Descriptive statistics, such as the frequencies, percentages, mean and standard deviations, were used to describe the demographic and work-related data.

**Figure 1:**
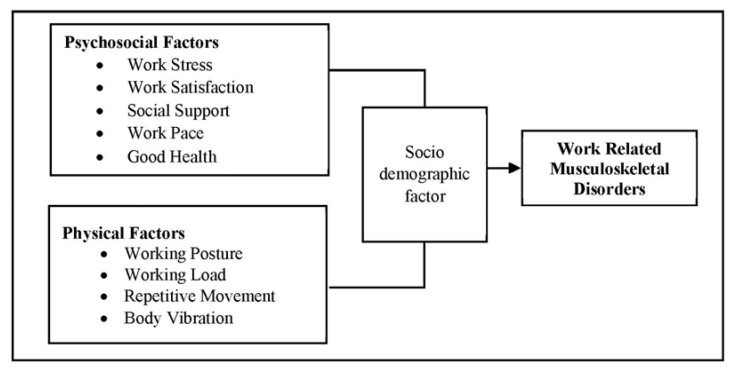
Conceptual framework.

Chi-square and logistic regression were used to determine the associations between some of the study variables and reported MSDs. The MSDs were assumed to be the dependent variables. Demographic characteristics and some of the workplace factors were defined as independent variables, as shown in Figure 1. A *p*-value < 0.05 was considered significant.

## 3. Results

In total, 303 questionnaires were distributed, and 300 nurses gave their consent to participate in the study; therefore, the response rate was 99.0%. Table 1 below shows the demographic information of the respondents.

### 3.1. Demographic Factors

The majority (187, 62.3%) of the respondents were 30–39 years old, indicating that most of the nurses were young. As per the WHO cut-offs for BMI, 136 (45.3%) nurses had a normal weight (BMI 18.5 to 24.9 kg/m^2^), meanwhile based on the Asia–Pacific cut-offs for BMI, 150 (50.0%) were classified as obese (BMI ≥ 25 kg/m^2^), as shown in Table 1 above. Surprisingly, 34% (102) of the nurses were classified as overweight (BMI 25–29.9 kg/m^2^) and 16% (48) were obese (BMI ≥ 30 kg/m^2^) based on the WHO guidelines, compared to 17.7% (53) of nurses who were overweight (BMI 23.0–24.9 kg/m^2^) and 50% (150) who were obese (BMI ≥ 25 kg/m^2^) based on the Asia–Pacific guidelines.

The majority (281, 93.7%) of nurses possessed a diploma in nursing, while 6.3% had a bachelor’s degree in nursing. In addition, 47.3% (142 nurses) were currently pursuing higher study part-time and the rest (52.7%) of the nurses remained at their previous academic qualifications. Regarding the years of experience, 39.0% (117 nurses) of the respondents had 5–10 years and 44.0% (132 nurses) had more than 10 years of nursing experience in public health services. The job tenure of a majority (83%) of the nurses was more or equivalent to 5 years of service.

### 3.2. Prevalence Findings Regarding WRMSDs for Different Body Regions

The self-reported prevalence rates (%) of musculoskeletal complaints or discomfort for 13 different parts of the body over 12 months among hospital nurses are shown in Table 2. More than three-quarters of the respondents had experienced symptoms in the lower back (86.7%), ankle/feet (86.7%), neck (86.0%), shoulder (85.3%), lower legs (85.0%), upper back (84.3%), knees (77.3%) and thighs (73.7%) in the previous 12 months. Moderate prevalence (less than 70%) of trouble was found for hip (66.0%) and forearm pain (61.7%). The parts of the body with lower prevalences of musculoskeletal complaints were wrist/hand (63.0%) and elbow (55.0%) discomfort/pain. In view of the prevalence rates, WRMSDs were estimated based on the pain occurrence frequency and intensity or degree of pain frequency at 13 body regions of the 300 participant nurses in the survey. The authors decided to focus on the top six body regions with a high prevalence (>80.0%) rate of WRMSDs for the analysis of the risk factor results. A high number of nurses (97.3%) complained of having work-related musculoskeletal pain during the previous 12 months.

#### Psychosocial Factor

**Figure 2:**
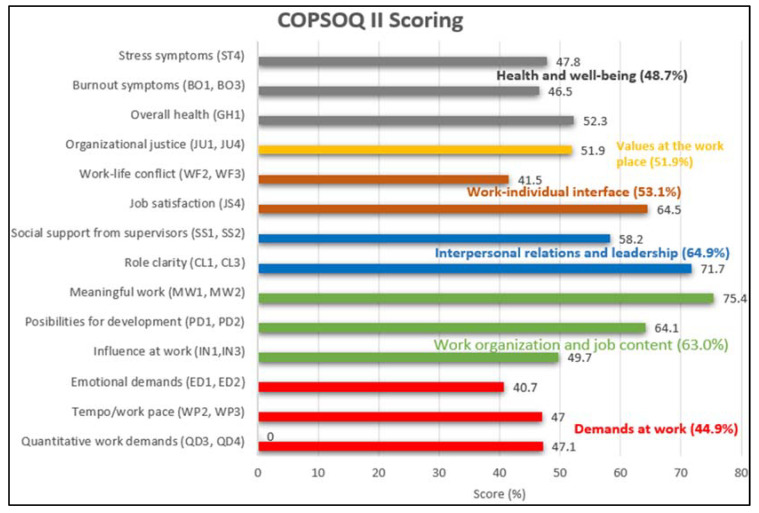
COPSOQ II results with 14 scales of psychosocial scoring for the nursing population.

The overall percentage of scoring value for the 14 scales of psychosocial factors (COPSOQ II), which consisted of the six main combined domains, were interpersonal relations and leadership (64.9%), work organization and job content (63.0%), work–individual interface (53.1%), values at the workplace (51.9%), health and well-being (48.7%) and demands at work (44.9%); these are shown in Figure 2 for the 300 nurse participants in the study. The work-related psychosocial factors with the highest scores (75.4, 71.7 and 64.5 on a 100-point scale) were meaningful work, followed by role clarity and job satisfaction respectively. The lowest scores (40.7 and 41.5 on a 100-point scale) were recorded for emotional demands and work–life conflicts among the participants, respectively.

The nurse participants from Hospital Sultan Abdul Halim (HSAH) had a better psychosocial working environment in terms of the meaning of work, role clarity, job satisfaction and possibilities for development and only in terms of emotional demands and work–life conflicts was the psychosocial working situation worse for them.

A high prevalence rate of lower back pain was obtained from research by Sheikzadeh et al. [27], who reported 84.0%, and Mirmohammadi et al. [28] reported an 89.1% prevalence rate of lower back among nurses in Isfahan, Iran. This may have been because of bending/twisting the back in awkward ways, standing for long hours when treating a large number of patients, inadequate breaks and lifting/transferring dependent patients. The lower back pain had a uniquely high prevalence rate among body parts; this high prevalence is common in nurses because of risk factors that they are exposed to, working in various environments and the job demands (psychosocial risk factors), such as stress [29,30,31].

### 3.3. Assessment of Occupational Job Tasks

Table 3 shows the percentages of the risk levels obtained based on the HIRARC 2008 in terms of the studied occupational job tasks among the nurses. More than 280 nurses (93%) were found to be at a medium risk level. These scores were obtained from an analysis of the job tasks listed in Table 4. These job tasks fell in the medium risk level as most of the participants nurses experienced a maximum level of discomfort (>20%) and sprain/strain symptoms (>23%) when a force was exerted on the body during job performance. The symptoms assessment from the work task were listed as no pain, pain, excess fatigue, sprain and strain, injury, discomfort and other symptoms by the participants to produce the risk level. The scores were obtained from job tasks such as sponging, dressing, pushing a bed, patient bed transfer and helping a patient walk. These job tasks required a maximum level of force to carry out, such as patient bed transfer, where the nurses needed to manually lift the patients, which included obese patients, who required more force to transfer them. The relative risk values fell within the medium category, as shown in Table 3 for all listed perceived job risk factors causing WRMSDs among nurses during their hospital duties. This requires a planned approach to controlling the hazards, including applying temporary measures if required.

## 4. Discussion

The high prevalence of MSDs among the nurses, particularly (97.3%) in this study, is worrisome as it may mean that the healthcare facility control measures against the risks of acquiring MSDs remain critical. This is also a grave concern because MSDs are the main contributing factors for an early exit from work and represent the most common cause of absenteeism among healthcare workers [33]. This finding is similar to a study done by Singh et al. [24], where the most common site affected was the lower back (36%), followed by the ankles/feet (26%), the neck (10%) and shoulders (8%). Another study done by Attar [34], in line with the present analysis results, found lower back and ankle/feet discomfort (88.7%), followed by neck (86.0%) and shoulder discomfort (85.3%), among the studied nursing population. The current study attempted to explore this association, which was significantly associated with the medical department, where nurses were more likely to have higher odds of upper back WRMSDs than nurses working in other departments. This study indicated that the physical workload varies between nurses depending on the nature of their wards and the variety of their task demands, which can consequently be specified as the frequency and duration of exposure to extreme postures. As shown in this research, most of the study participants were exposed to frequent high-risk and complex tasks, although the portion of time spent in extreme postures varied according to their wards. This may not be considered surprising given the variety of physically demanding tasks that are given to hospital nurses when undertaking their routine activities. Nurses from SCN (pediatrics) and orthopedic wards were less likely (lower odds) (OR: 0.13, 95% CI: 0.33–0.55, *p* = 0.006 and OR: 0.20, 95% CI: 0.05–0.87, *p* = 0.031, respectively) to have upper back pain as compared to nurses in the medical ward. The results indicated that awkward posture and increased physical workload among nurses working in the medical, orthopedic and pediatrics wards was generally higher than for other participants. According to [35], nurses working in the emergency department showed the highest prevalence of WRMSDs among nurses since they were involved in frequent lifting and pushing in the clinical practice.

In another supportive study regarding the effect of the department/ward, operation room nurses had the highest (74.3%) prevalence rate WRMSDs in terms of discomfort incidence because of the peculiarity of the job tasks undertaken throughout the surgery process [33]. Some epidemiologic studies indicated an association between occupational factors and WRMSDs. Further, some reported that the prevalence and location of pain, along with other symptoms, can be related to the standing posture, work habits and other demographic characteristics [36]. Logistic regression analysis revealed a significant relationship between job tenure and pain in the ankles/feet, neck, shoulders and lower legs (*p* < 0.05). In other studies, the rate of WRMSDs significantly increased with increasing work experience, which is similar to the results obtained in our study [5].

Nurses working for more than seven hours per day for continuous six days (>42 h) were less likely (lower odds) to have lower back and shoulder pain as compared to those who worked for six days at seven hours per day (42 h) in the current study, which is consistent with the result of Koohpayehzadeh et al. [37] for shoulder pain in 512 nursing personnel in the Iran Medical University of Medical Sciences in Tehran and the findings of [38] regarding low back pain. In contrast with our study results, other studies found that there was no significant association between working hours and MSD pain [3,39,40].

## 5. Limitations

This study yielded important results on the prevalence and risk factors of WRMSDs among nurses in Malaysia. However, it had a few limitations. First, the results were from a single institution. Thus, future studies should be involved with several institutions to increase the generalizability. This study had a possible response bias, as the questionnaires were distributed and collected by the nurse managers of the various wards and units from which the participants were selected. However, the researchers believe that any coercive pressure arising from the nurse managers as go-betweens was negated by the instructions given to the respondents to seal the completed questionnaires in the envelopes provided before returning them to the nurse managers. This, included with the assignment of a unique code to each respondent, ensured confidentiality and anonymity.

## 6. Conclusions

This study showed a high prevalence of WRMSDs in nurses (97.3%), with the highest prevalence rates being for low back and ankle/feet pain, followed by neck and shoulder pain, whereas the prevalence rate for elbow pain was lowest among the thirteen body regions covered by the study. In this study, the major risk factors that were responsible for MSDs were age, lower education level, female gender, high BMI and job tenure. These association patterns also suggest prospects for intervention strategies to stimulate healthy lifestyles and increase a positive psychosocial work environment. Postural education should be provided to nurses by encouraging short breaks. Proper screening programs should be conducted regarding the risk factors of musculoskeletal pain in nurses, and prevention, early diagnosis, treatment, rehabilitation and counseling are necessary. In addition, nurses with MSD issues might be helped by increasing the number of employees and having their working hours rescheduled by the healthcare manager or top management.

## Figures and Tables

**Table 1:** Socio-demographic characteristics.

Variables	Frequency	Percentage
Female	300	100.0
Age group		
23–29 years	40	13.3
30–39 years	187	62.3
40–49 years	57	19.0
50–55 years	16	5.3
Academic qualification		
Diploma	281	93.7
Degree	19	6.3
Currently pursuing higher studies		
Yes	142	47.3
No	158	52.7
Married	252	84.0
No child	72	28.6
Single child	25	9.9
2 children	59	23.4
3 children	51	20.2
4 children	34	13.5
5 children	10	4.0
6 children	1	0.4
Single	16	5.3
Widow	8	2.7
Separated/divorced	24	8.0
Job tenure		
Less than 3 months	3	1.00
3 months to 1 year	7	2.33
1 year to 5 years	41	13.67
5 years to 10 years	117	39.00
More than 10 years	132	44.00
BMI (WHO classification) (kg/m^2^)		
Underweight (<18.5)	14	4.7
Normal (18.5–24.9)	136	45.3
Overweight (25–29.9)	102	34.0
Obese (30 and above)	48	16.0
BMI (Asia–Pacific classification) (kg/m^2^)		
Underweight (<18.5)	14	4.7
Normal (18.5–22.9)	83	27.7
Overweight (23–24.9)	53	17.7
Obese (25 and above)	150	50.0

**Table 2:** Prevalence of WRMSDs.

No.	Body Regions	No. (Proportion)	Proportion Confidence Interval (95% CI)
1.	Lower back	260 (86.7%)	82.8–90.5%
2.	Ankles/feet	260 (86.7%)	82.8–90.5%
3.	Neck	258 (86.0%)	82.1–89.9%
4.	Shoulders	256 (85.3%)	81.3–89.4%
6.	Lower legs	255 (85.0%)	80.9–89.1%
7.	Upper back	253 (84.3%)	80.2–88.5%
8.	Knees	232 (77.33%)	72.6–82.1%
9.	Thighs	221 (73.7%)	68.7–78.7%
10.	Hips	198 (66.0%)	60.6–71.4%
11.	Wrist/hand	189 (63.0%)	57.5–67.2%
12.	Forearms	185 (61.7%)	56.1–67.2%
13.	Elbows	165 (55.0%)	49.3–60.7%

**Table 3:** List of job tasks undertaken by nurses at the hospital with their WRMSD symptom frequencies.

No.	Job Task	Frequency	Percentage
1.	Repetitive motion		
	No risk	8	2.67
2.	Body vibration		
	No risk	22	7.3
	Medium risk	278	92.67

**Table 4:** Risk assessment based on the HIRARC guidelines [32].

No.	Effects Based on Hazards during Job Task	Frequency	Percentage
1.	Repetitive motion		
	No pain	8	2.67
	Pain	60	20.00
	Excess fatigue	48	16.00
	Sprain and strain	64	21.33
	Injury	26	8.67
	Discomfort	65	21.67
	Others	29	9.67
2.	Whole-body vibration		
	No pain	22	7.33
	Pain	63	21.00
	Excess fatigue	37	12.33
	Sprain and strain	59	19.67
	Injury	9	3.00
	Discomfort	67	22.33
	Others	43	14.33

## Data Availability

The data presented in this study are available from the corresponding author on request.
